# Investigating Pathways to Minimize Sensor Power Usage for the Internet of Remote Things

**DOI:** 10.3390/s23218871

**Published:** 2023-10-31

**Authors:** Tiana Cristina Majcan, Solomon Ould, Nick S. Bennett

**Affiliations:** 1 Centre for Advanced Manufacturing, University of Technology Sydney, Broadway, Ultimo, Sydney, NSW 2007, Australia; tiana.majcan@uts.edu.au (T.C.M.); solomon.ould@uts.edu.au (S.O.); 2 Radio Frequency and Communication Technologies Laboratory, University of Technology Sydney, Broadway, Ultimo, Sydney, NSW 2007, Australia

**Keywords:** Internet of Remote Things, sensor, low power

## Abstract

The Internet of Remote Things (IoRT) offers an exciting landscape for the development and deployment of remote wireless sensing nodes (WSNs) which can gather useful environmental data. Low Power Wide Area Networks (LPWANs) provide an ideal network topology for enabling the IoRT, but due to the remote location of these WSNs, the power and energy requirements for such systems must be accurately determined before deployment, as devices will be running on limited energy resources, such as long-life batteries or energy harvesting. Various sensor modules that are available on the consumer market are suitable for these applications; however, the exact power requirements and characteristics of the sensor are often not stated in datasheets, nor verified experimentally. This study details an experimental procedure where the energy requirements are measured for various sensor modules that are available for Arduino and other microcontroller units (MCUs). First, the static power consumption of continually powered sensors was measured. The impact of sensor warm-up time, associated with powering on the sensor and waiting for reliable measurements, is also explored. Finally, the opportunity to reduce power for sensors which have multiple outputs was investigated to see if there is any significant reduction in power consumption when obtaining readings from fewer outputs than all that are available. It was found that, generally, CO_2_ and soil moisture sensors have a large power requirement when compared with temperature, humidity and pressure sensors. Limiting multiple sensor outputs was shown not to reduce power consumption. The warm-up time for analog sensors and digital sensors was generally negligible and in the order of 10–50 ms. However, one CO_2_ sensor had a large overhead warm-up time of several seconds which added a significant energy burden. It was found that more, or as much, power could be consumed during warm-up as during the actual measurement phase. Finally, this study found disparity between power consumption values in datasheets and experimental measurements, which could have significant consequences in terms of battery life in the field.

## 1. Introduction

The Internet of Remote Things (IoRT) is a rapidly expanding area of Internet of Things (IoT) technology, which utilizes long range, low power networks (LPWANs) to communicate. While IoT traditionally relies on the use of high data rate public networks, such as those devices which are deployed in cities, homes and industry, IoRT enabled using LPWANs has become popular due to the constraints of collecting various data in remote locations [1,2]. As a result, LPWANs are now widely used for data gathering in hostile or remote locations, and the observation of such data can substantially improve our comprehension of complex ecological and climate research [3,4,5,6,7]. LoRa (short for Long Range) is a particular LPWAN protocol which is well suited for power constrained devices, which transmit small data packets. Communication over LoRa can achieve a very low power consumption for transmission of data up to 20 km; however, models and experimental research show that even further transmission, e.g., to low orbit satellites, may be viable [5,8,9]. This provides promise for the deployment of highly remote devices which can provide useful environmental data—often referred to as Wireless Sensor Nodes (WSNs)—and would have otherwise required a nearby network infrastructure to be enabled.

### 1.1. Internet of Things in Remote Areas

The IoRT is generally enabled by either satellites or unmanned aerial vehicles (UAVs), or combinations of both [6,7]. Satellite-based internet connection in remote areas is a popular method which has been gaining traction [3,9]. This is due in part to ubiquitous service, as well as the falling cost of satellite services compared with fibre or broadband alternatives, and several satellite enabled IoRT networks have been demonstrated. Chen et al. [3] illustrates a case in which environmental data were collected across the remote and hostile areas of the Tibetan Plateau, which cannot access public communication networks. Their paper shows evidence of a high-quality implementation which successfully monitored a variety of environmental metrics such as precipitation, wind speed, snow fall, etc. However, Gaggero et al. [6] and Zhang et al. [7] argue that a satellite enabled IoRT has several limitations which can be addressed with UAVs in combination with satellites. Even in the most remote places, satellites can provide connection; however, high bandwidth data transfer, e.g., for photos and video, currently remains expensive by satellite and needs additional improvement to be practical.

### 1.2. Power Requirements for LoRa Prototype Boards

A comprehensive model for the energy consumption of wireless sensor nodes deployed using LoRaWAN was investigated by Bouguera et al. [10]; they provided estimates to determine the sensor lifetime and optimise power consumption. They also found that the energy consumption is dependent on various LoRaWAN factors such as range, spreading factor and transmission power, which must be carefully chosen for optimisation.

Experimental validations in literature also align with the findings of Bouguera et al., where researchers found message transmission events to be the main contributor to the power consumption of these devices [11,12,13]. Casals et al. [14] provide a model for the current consumption for various popular LoRa radio chips, which has been validated by Bouguera et al. However, this did not include models for the processing units or sensors, therefore it is not indicative of real use cases. Ould and Bennett [12] have outlined how the manufacturers of prototype boards containing a LoRa radio and Micro Controller Unit (MCU) marketed as “low power” only detail the deep sleep power consumption of the board and overlook other important functions associated with LoRA communication. Their experimental findings showed that the power consumption across multiple prototype boards on the market for the same routine differs by up to four times. Over the course of a device’s deployed lifetime, this accumulative power consumption would significantly impact the energy requirements for these boards. Ould and Bennett also developed a model for LoRa boards alongside sensors and conducted preliminary experiments, finding that the sensor power draw remained relatively constant and was accurate to the model. The effects of a temporal compression algorithm on reducing transmission periods for LoRa IoT devices was investigated by Väänänen and Hämäläinen [15]. They experimentally measured the power draw of a LoRa device in conjunction with a DHT22 temperature and humidity sensor and the effects of the temporal algorithm. While their conclusions are focused on the algorithm’s impact, they found that the average power draw for the DHT22 sensor was 5 mW without using compression techniques. Even so, there are few previous works which experimentally validate the power consumption of a LoRa radio and an MCU alongside various available environmental sensor modules.

Energy harvesting and high-capacity batteries are potential solutions for powering remote devices to minimize maintenance and labour. Cheong et al. [16] presented findings that showed ultra-low power devices with less frequent communications may simply be powered by a single battery for over 10 years. Battery operation poses significant benefits to energy harvesting solutions such as solar power. Ould and Bennett [12] also note that sensors such as thermocouples or CO_2_ detectors will require a certain “warm-up” time, so they need to be powered on before being able to take an accurate reading, which will affect the power consumption of such sensors. While Bennett and Ould have outlined the power requirements of a small selection of sensors over a 30 s duty cycle, their paper is explicit in stating that more comprehensive modelling accompanied by experimental validation is required due to the lack of manufacturer data for various sensing tasks that better represent the use cases in IoRT.

### 1.3. Pathways to Minimize Power Usage

The need for remote, low-powered, low-maintenance IoT-enabled devices has grown in demand and is documented in the scientific literature. Due to the convenience and safety of having devices collect and transmit data from remote locations, the exact power requirements for such devices are exceedingly important, as energy sources must be well equipped to last many years or indefinitely [17]. However, as this area of research is still in its infancy, the exact power requirements of various prototyping boards with MCUs alongside sensor modules or WSNs is still unknown, and, if stated in the manufacturer specification, lack sufficient detail. The goal of this study was to quantitatively assess the power requirements of sensor modules which are capable of measuring multiple environmental parameters, such that the energy requirements of WSNs can be better estimated. This minimizes the maintenance, cost and possibly dangerous labour hours associated with the deployment of remote devices and WSNs.

Following on from the research conducted by Ould and Bennett [12], the following research questions were explored using empirical data gathered from experimentation:

1. Which consumer available sensor modules, that can sense the following environmental metrics, have the lowest power draw in conjunction over a fixed duty cycle and identical firmware routine:
  - Temperature
  - Humidity
  - Soil moisture
  - Soil pH
  - CO_2_
2. For sensors which have multiple outputs, is there any significant reduction in power consumption when obtaining readings from fewer outputs than all that are available?
3. What are the associated (if any) warm-up times for sensors in the above categories, and what is the minimum time delay/power consumption before a reading can be obtained?

## 2. Materials and Methods

The aim of this study was to gather quantitative data, experimentally, on the power draw of various sensor modules in conjunction with various development boards. This was to determine which sensors would be best suited for a remote device application or as WSNs, and accurately determine the energy requirements for such a remote device so that appropriate energy harvesting techniques or battery capacity can be chosen. Additionally, sensors which have multiple sensor outputs generally communicate via serial communication buses, such as I2C or 1-Wire, do not have clear energy requirements.

Table 1 shows the characteristics of the sensors which were obtained for this study. The table contains a description of what each sensor can detect, as well as the connection interface, operating voltage and the stated current draw, taken from the manufacturer’s data sheet. Many of these sensors can sense multiple parameters and are all marketed as being “low powered” or “low-cost”, or a combination of these factors. The sensors were chosen as likely sensors which may be desirable for remote sensing applications in the IoRT.

For all experiments, a Keithley 2460 SourceMeter was used in 2 wire sense mode, acting as an ammeter in series with the device under test (DUT)—in this case, the sensor module. This experimental circuit differed from the work conducted by Ould and Bennett [12] in that the sensors were not powered by the Keithley 2460 and instead used the 5 V or 3.3 V rail onboard the MCU. An Arduino Leonardo was used as the primary MCU for powering the sensors and obtaining sensor data, alongside a low power Grasshopper Lora board (refer to Table 2 and Table 3 for specifications).

The data from the Keithley 2460 SourceMeter contained time-stamped current data ranging between 5 and 15 samples a second. For each sensor, the current data was recorded and then the average, peak and, if applicable, idle current draw, and hence power consumption, could be determined. The mean power draw of each sensor was calculated using the mean current draw of the sensor over 60 s (*I*) and using the formula for electric power (*P* = *IV*) where *V* is sensor input voltage, either 3.3 V or 5 V.

### 2.1. Continually Powered Sensors

A basic firmware routine for each sensor was prepared which powered on the sensor and, in the case of digital sensors, established a connection to the sensor. Sensor readings were then requested via USB serial routinely at 5 s intervals, to allow ample time for us to recognize power consumption responses in order to detect patterns associated with accessing sensor data. For modules which had multiple sensors on board, all available sensor outputs were requested. Although accuracy of sensor readings is not within the scope of this project, the sensor values were output into a serial monitor to ensure they were within sensible limits and, in the case of digital I2C or 1-Wire sensors, that there was no corruption to the bus.

### 2.2. Multi Variable Sensor Comparison

For I2C sensors which provide multiple outputs, a similar procedure was conducted to determine the difference (if any) in the power consumption of the sensor when reading one versus multiple outputs. The sensors used for this analysis are described in Table 4. For each sensor a basic firmware, which requested only one or combinations of two sensor outputs (i.e., less outputs than are available from the sensor) at 5 s intervals, was created for the Arduino Leonardo. The current in the sensor power circuit was recorded for each combination of variables for comparison using the Keithley 2460 SourceMeter.

### 2.3. Warm-Up Time

As shown in previous literature, some sensors require a warm-up before being able to provide accurate sensor data [12]. This experiment aimed at determining the power consumption associated with the minimum amount of time taken for a sensor to be powered before being able to obtain an accurate reading. Many electrochemical sensors, such as those used in CO_2_ sensing modules, are required to have a “burn in” time to calibrate the sensor for accurate sensing. While this is important to consider in the applications that are common for remote sensing, it will not be treated as warm-up time in this case, since it is a one-time activity that does not need to be repeated every time the sensor is powered.

Firmware was created for the Arduino Leonardo which powered-on the sensor and requested data readings every 50 ms as soon as the sensor was available. For multi variable sensors, all possible combinations of variables were obtained. The current draw of the sensor module was recorded using the Keithley 2560 SourceMeter, which started recording from when the microcontroller was being held in “reset” state and recorded for the duration of the microcontroller and sensor start-up routine. The sensor reading was output via USB serial with time-stamped values, such that the sensor values could be matched in time with the current readings from the SourceMeter. The sensors used for this analysis are described in Table 5.

## 3. Results

### 3.1. Continually Powered Sensors

**Figure 1:**
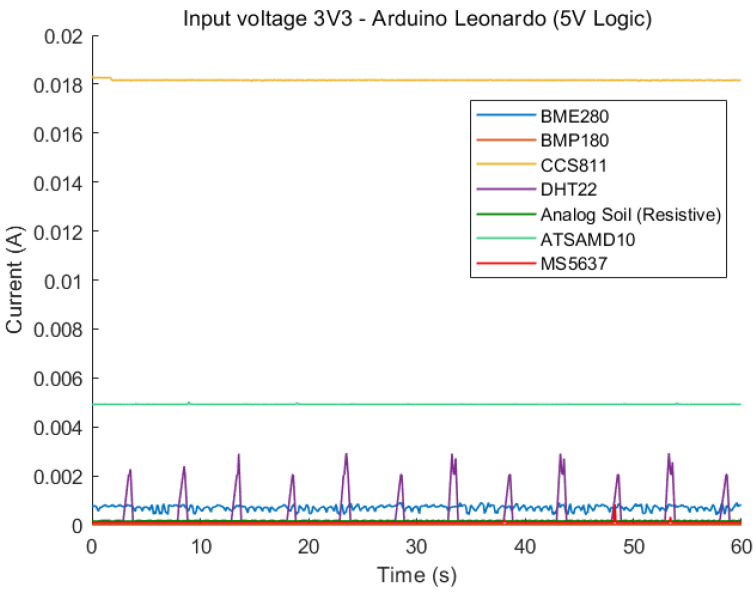
Comparison of static current draw of all sensors, input voltage is 3.3 V.

**Figure 2:**
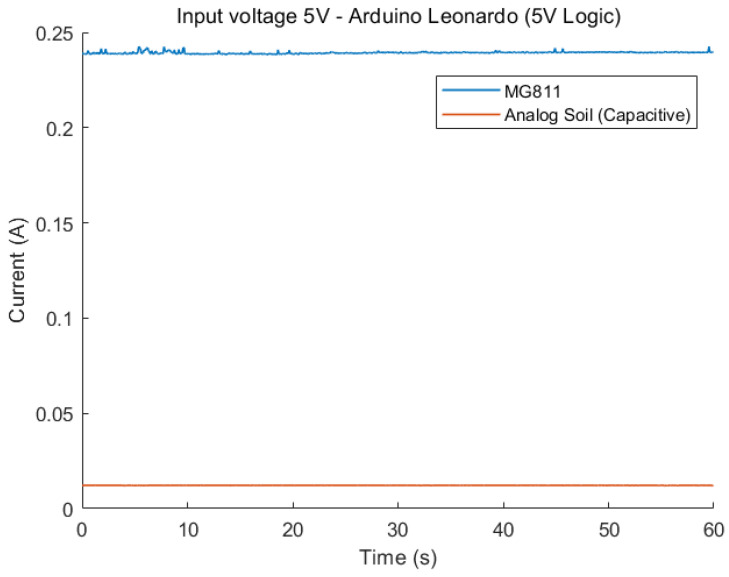
Comparison of static current draw of all sensors, input voltage is 5 V.

The current draw of all measured sensors is shown in Figure 1 and Figure 2. As expected, the majority of sensors are relatively low-power compared to the CCS811 and MG811 CO_2_ sensors, with the MG811 having the highest overall current consumption. 

**Figure 3:**
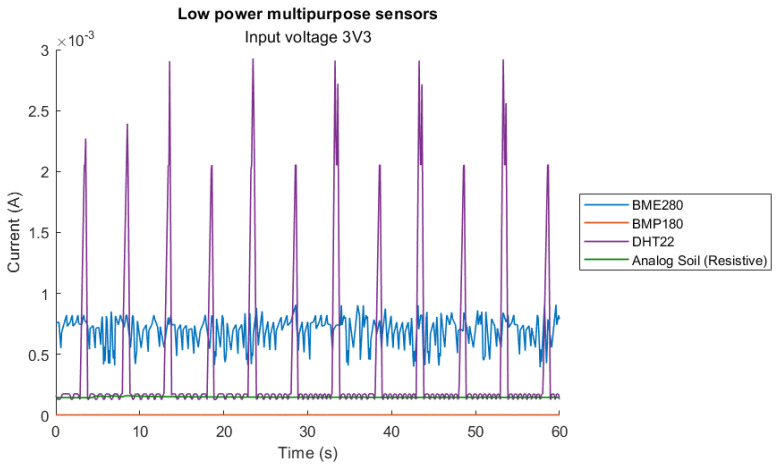
Detailed current draw for lower current sensors.

Similarly, the peak current is determined by obtaining the maximum current value over the 60 s period and determining the associated peak power value. The results are shown in Table 6. A closer look at the lower powered sensors can be seen in Figure 3. The DHT22 has clear peaks at 5 s intervals, which correlates exactly with the interval at which the firmware requests sensor values. The peaks represent a significant power requirement when reading sensor data from the DHT22, where the power consumption is up to 10 times that of the average idle consumption while powered. Compared with the BME280, analog soil and BMP180 sensors, the DHT22 is the only sensor which shows noticeable peaks at 5 s intervals, which are associated with the requesting of data from the MCU.

### 3.2. Multi Variable Power Consumption

**Figure 4:**
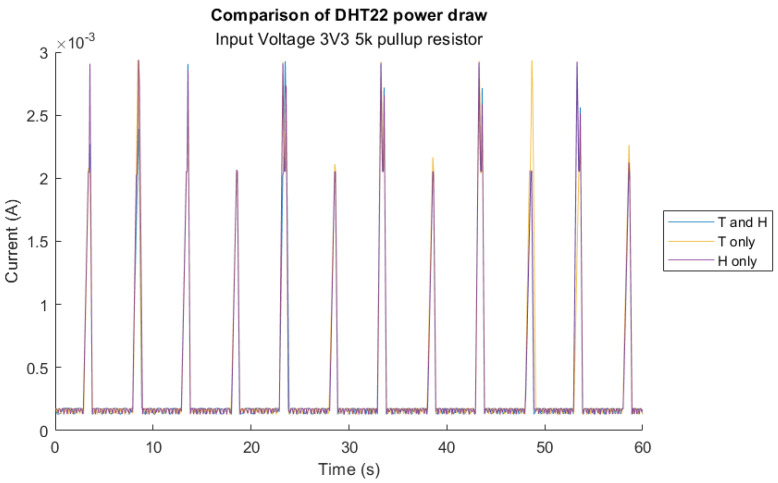
DHT22 current draw when requesting all or only individual sensor variable where T = temperature, H = humidity.

**Figure 5:**
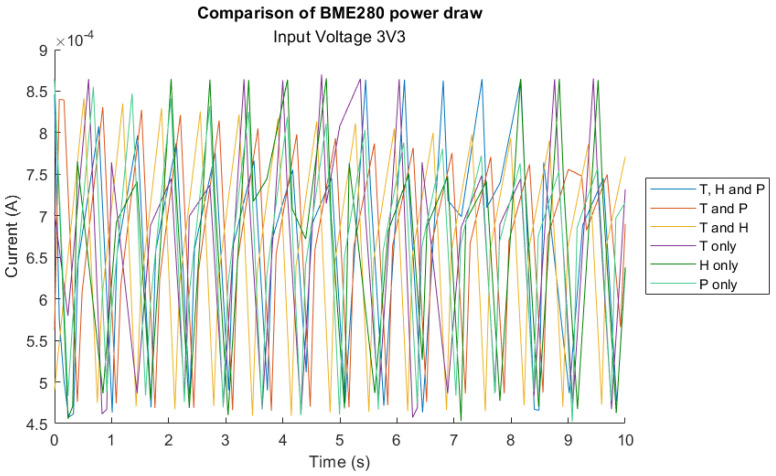
BME280 current draw when requesting all or combinations of fewer sensor variables where T = temperature, H = humidity, P = pressure.

The current draw for the DHT22 and BME280 sensors while measuring one or more combinations of outputs is shown in Figure 4 and Figure 5. The average current and power consumption for each output or combination of outputs are outlined in Table 7 and Table 8.

While the DHT22 uses a 1-Wire interface to send data to the MCU and the BME280 interfaces with I2C, there appears to be no discernible difference when requesting all or only one available sensor output.

### 3.3. Warm up Time

**Figure 6:**
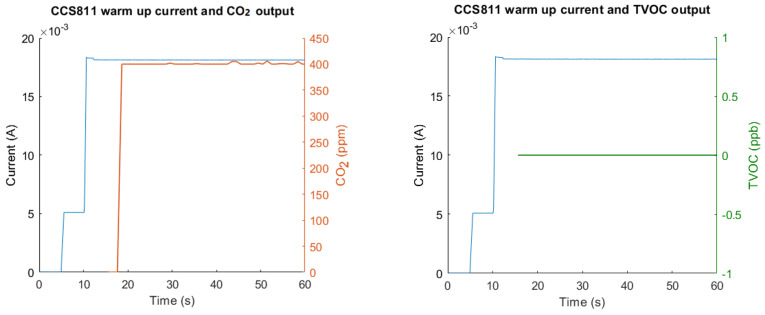
CCS811 sensor warm-up current with CO_2_ (**left**) and TVOC (**right**) output.

The warm-up time is determined by the minimum amount of time between powering a sensor and the output of reliably measured data. The warm-up energy consumption is the energy consumed by the sensor during the warm-up time. Other than CCS811, once initialized all sensors could output data within a relatively short time span, generally 10–20 ms. The energy ‘wasted’ for warm-up is therefore negligible. However, CCS811 took several seconds from the point of initialization to the output of sensible values, as shown in Figure 6. By integrating the area under the blue line in Figure 6 from 0 s to 18.1 s, and multiplying by the input voltage (3.3 V), the energy consumption during warm-up was derived as 0.86 J. 

## 4. Discussion

### 4.1. Overall Current Consumption

From the data gathered across all sensors, it was evident that there are many suitable low-power options which have a short warm-up time and can be powered on and provide readings within a few milliseconds. The CO_2_ sensors tended to have the highest power consumption, which is in line with their respective datasheets. Second to that, soil moisture sensors, which are dependent on resistivity of the moisture content of the soil (i.e., how much water is in the soil) had current consumptions which varied by orders of magnitude across that sensor category. For the resistive analog soil moisture sensor, the power consumption was linearly related to the moisture content of the soil—up to a maximum of 0.24 mA or 0.792 mW when submerged in 100% water. The capacitive soil sensor did not vary in power consumption in this way.

The DHT22 is an example of a sensor which, while powered, will draw an idle current that is very low at approximately 0.015 mA, but will have a large spike of up to 10 times the idle current at the point of reading values.

### 4.2. Multi-Variable Sensors

Digital sensors which provide multiple outputs can be a highly efficient way to gather multiple environmental metrics while maintaining an overall low system power requirement. However, as evidenced by Figure 4 and Figure 5, the sensor prepares all sensor outputs regardless of whether they are requested by the microcontroller, and, therefore, the power consumption remains the same regardless of reading only one versus all outputs. Interestingly, the BME280 datasheet explicitly states the measuring current draw, however the experimental values do not match as they are significantly higher. Although the comparison between sensors which provide multiple outputs and those which only measure a single metric could not be obtained, this finding has strong implications for the future development of WSNs which use digital sensors. It is imperative that for WSNs deployed in IoRT that sensors are carefully chosen to provide only the sensor outputs that are needed, in order to avoid wasted energy consumption.

### 4.3. Warm-Up Time

For most sensors, besides the CCS811 CO_2_ sensor, warm-up time is negligible and in the order of 10–20 ms. The CCS811 took a total of 18 s between initialization and being able to output any sensor values, and which equates to a total fixed warm-up energy consumption of 0.86 J. Given the peak power consumption of this sensor is 60 mW (Table 6), this equates to the same amount of energy that is consumed during approximately 14 s of ‘peak’ operation. Since it is viable that a one-off CO_2_ measurement could be made in 14 s or less, it is therefore likely that more, or as much, power could be consumed during warm-up as during the actual measurement phase. This associated warm-up time, especially for measuring CO_2_, is therefore crucial in determining the overall power consumption of WSNs, particularly when it comes to predicting battery lifetime or sizing a battery to last for a desired duration. Likewise, while the datasheet specifies minimum times for the start-up routine of the sensor, this value is significantly lower than that which was determined experimentally.

## 5. Conclusions

The Internet of Remote Things offers exciting potential for the development and de-ployment of remote wireless sensing nodes, which can gather useful environmental data. Low Power Wide Area Networks provide an ideal network topology for enabling the IoRT; however, due to the remote location of these WSNs, the power and energy requirements for such systems must be accurately determined before deployment, as devices will be running on limited energy resources, such as long-life batteries or energy harvesting. In this study the power consumption of various sensor modules which are available on the consumer market has been empirically investigated. This was undertaken as the precise power needs of these sensors are frequently not mentioned in datasheets, despite the fact that many of these sensors are suitable for usage in LPWAN and IoRT applications where accurate power information is vital. This study found disparity between the power consumption values reported in datasheets and the experimental measurements, which could have significant consequences in terms of battery life in the field. The static power consumption of continually powered sensors, as well as the warm-up time and fixed energy consumption associated with powering on the sensor and taking a measurement, was explored. From empirical data the average and peak power consumption is calculated using current sensing data gathered from each sensor. It was found that, generally, CO_2_ and soil moisture sensors have a large power requirement when compared with temperature, humidity and pressure sensors. The warm-up time for analog sensors and digital sensors which did not sense CO_2_ was generally negligible and in the order of 10–50 ms. However, the CCS811 CO_2_ sensor had a large overhead warm-up time of several seconds, and an associated power consumption of 0.86 J before sensible readings could be taken. It is therefore likely that more, or as much, power could be consumed during warm-up as during the actual measurement phase. The findings of this study into powering remote sensors have strong implications for applications within IoRT and specifically LPWANs, laying the foundations for future research and empirically verified baseline power consumption for the development of WSNs.

## Figures and Tables

**Table 1:** List of sensors. Stated current draw taken from datasheets [18,19,20,21,22,23,24,25,26].

Chip Serial	Description	Interface	Operating Voltage	Stated Current Draw
BME280	Temperature, Humidity and Pressure Sensor	I2C or SPI	3.3 V	3.6 μA *
DHT22	Temperature and Relative Humidity Sensor	1-Wire	3.3 V	1–1.5 mA
CSS811	CO_2_ and Temperature Sensor	I2C	3.3 V	30 mA
MG811	CO_2_ Sensor	Analog	5 V	N/A
N/A	Soil Moisture Sensor	Analog	3.3 V	N/A
ATSAMD10	Capacitive Soil Moisture Sensor	I2C	3.3 V	N/A
N/A	Capacitive Soil Moisture Sensor	Analog	5 V	N/A
BMP180	Pressure Sensor Module	I2C	3.3 V	5 μA
MS5637	Pressure Sensor Module	I2C	3.3 V	0.6 μA

* Datasheet also states that consumption is 1.8 μA when measuring just humidity and temperature or 2.8 μA when measuring just pressure and temperature.

**Table 2:** Arduino Leonardo specifications [27].

Feature	Value
Processor	ATmega32U4
Clock Speed	16 MHz
Logic Level	5 V
SPI	Yes
I2C	Yes
UART	Yes
Output Voltages	5 V, 3V3

**Table 3:** Grasshopper specifications [28].

Feature	Value
Processor	STM32L082
Clock Speed	32 MHz
Logic Level	3V3
SPI	Yes
I2C	Yes
UART	Yes
Output Voltage	3V3
Lora Radio	SX1276

**Table 4:** Multi variable sensors used for current draw differences between reading all or one variable and which environmental parameters they are capable of sensing.

Chip Serial	Temperature	Humidity	Pressure	Soil Moisture	CO_2_
DHT22	X	X			
BME280	X	X	X		

**Table 5:** Sensors used to investigate power consumption associated with sensor warm up time. All sensors used I2C interface.

Chip Serial	Temperature	Humidity	Pressure	Soil Moisture	CO_2_
BME280	X	X	X		
CCS811	X				X
DHT22	X	X			
MS5627	X		X		

**Table 6:** Average and peak current and power for 3.3 V and 5 V sensors (Sensors are 3.3 V unless stated in parentheses).

	Average Current (mA)	Average Power (mW)	Peak Current (mA)	Peak Power (mW)
BME280	0.67	2.22	0.91	2.99
BMP180	0.0038	0.013	0.0050	0.017
CCS811	18.17	59.95	18.33	60.49
DHT22	0.29	0.96	2.93	9.66
Analog Soil (Resistive)	0.15	0.50	0.16	0.54
ATSAMD10	4.92	16.24	5.026	16.59
MS5637	0.097	0.32	0.84	2.76
MG811 (5 V)	239.31	789.72	242.66	800.79
Analog Soil (Capacitive) (5 V)	12.19	40.23	12.22	40.32

**Table 7:** Average current and power consumption of DHT2 when requesting all or singular sensor outputs. T = temperature, H = humidity.

DHT22	Average Current (mA)	Average Power (mW)
T and H	0.30	0.99
T only	0.30	0.98
H only	0.31	1.02

**Table 8:** Average current and power consumption of BME280 when requesting all or singular sensor outputs. T = temperature, H = humidity, P = pressure.

BME280	Average Current (mA)	Average Power (mW)
T, H and P	0.68	2.25
T and P	0.67	2.22
T and H	0.68	2.25
T only	0.68	2.25
H only	0.68	2.25
P only	0.68	2.23

## Data Availability

Data from the study are available from the corresponding author by request.

## References

[1] Petäjäjärvi J., Mikhaylov K., Pettissalo M., Janhunen J., Iinatti J. (2017). Performance of a low-power wide-area network based on LoRa technology: Doppler robustness, scalability, and coverage. Int. J. Distrib. Sens. Netw..

[2] Zhang M., Li X. (2020). Drone-Enabled Internet-of-Things Relay for Environmental Monitoring in Remote Areas Without Public Networks. IEEE Internet Things J..

[3] Chen Y., Zhang M., Li X., Che T., Jin R., Guo J., Yang W., An B., Nie X. (2022). Satellite-Enabled Internet of Remote Things Network Transmits Field Data from the Most Remote Areas of the Tibetan Plateau. Sensors.

[4] Du X., Ma H. (2022). Eco-Environmental Civilization Construction System in Remote Areas Based on Multiple Data Collection and the Internet of Things. J. Sens..

[5] Wong A., Chow Y.T. (2020). Solar-supplied satellite internet access point for the internet of things in remote areas. Sensors.

[6] Gaggero G.B., Marchese M., Moheddine A., Patrone F. (2021). A Possible Smart Metering System Evolution for Rural and Remote Areas Employing Unmanned Aerial Vehicles and Internet of Things in Smart Grids. Sensors.

[7] Zhang M., Zhang L., Zhao C., Jin R., Guo J., Li X. (2022). Fetching Ecosystem Monitoring Data in Extreme Areas via a Drone-Enabled Internet of Remote Things. IEEE Internet Things J..

[8] Kiki M.J.M., Iddi I. (2022). Improved LORA Modulation Output in LEO Satellite Internet of Things. J. Electr. Eng. Technol..

[9] Lysogor I., Voskov L., Rolich A., Efremov S. (2019). Study of Data Transfer in a Heterogeneous LoRa-Satellite Network for the Internet of Remote Things. Sensors.

[10] Bouguera T., Diouris J.F., Chaillout J.J., Jaouadi R., Andrieux G. (2018). Energy Consumption Model for Sensor Nodes Based on LoRa and LoRaWAN. Sensors.

[11] Bäumker E., Garcia M., Woias P. (2019). Minimizing power consumption of LoRa^®^ and LoRaWAN for low-power wireless sensor nodes. J. Phys. Conf. Ser..

[12] Ould S., Bennett N.S. (2021). Energy Performance Analysis and Modelling of LoRa Prototyping Boards. Sensors.

[13] Singh R.K., Puluckul P.P., Berkvens R., Weyn M. (2020). Energy Consumption Analysis of LPWAN Technologies and Lifetime Estimation for IoT Application. Sensors.

[14] Casals L., Mir B., Vidal R., Gomez C. (2017). Modeling the energy performance of LoRaWAN. Sensors.

[15] Väänänen O., Hämäläinen T. (2022). Efficiency of temporal sensor data compression methods to reduce LoRa-based sensor node energy consumption. Sens. Rev..

[16] Cheong P.S., Bergs J., Hawinkel C., Famaey J. Comparison of LoRaWAN Classes and their Power Consumption. Proceedings of the IEEE Symposium on Communications and Vehicular Technology (SCVT).

[17] Augustin A., Yi J., Clausen T., Townsley W.M. (2016). A Study of LoRa: Long Range & Low Power Networks for the Internet of Things. Sensors.

[18] Adafruit (2018). Adafruit STEMMA Soil Sensor—I2C Capacitive Moisture Sensor. https://learn.adafruit.com/adafruit-stemma-soil-sensor-i2c-capacitive-moisture-sensor.

[19] ams (2016). CCS811 Datasheet. https://cdn.sparkfun.com/assets/learn_tutorials/1/4/3/CCS811_Datasheet-DS000459.pdf.

[20] Aosong Electronics Co., Ltd. Digital-Output Relative Humidity & Temperature Sensor/Module DHT22 (DHT22 Also Named as AM2302) Capacitive-Type Humidity and Temperature Module/Sensor DHT22. https://www.sparkfun.com/datasheets/Sensors/Temperature/DHT22.pdf.

[21] Bosch (2013). BMP180 Digital Pressure Sensor. https://cdn-shop.adafruit.com/datasheets/BST-BMP180-DS000-09.pdf.

[22] Bosch (2018). BME280-Data Sheet. https://www.mouser.com/datasheet/2/783/BST-BME280-DS002-1509607.pdf.

[23] Core Electronics Australia (2023). Capacitive Soil Moisture Sensor (Analog Output). https://core-electronics.com.au/soil-moisture-sensor-capacative-analog.html.

[24] Sandbox Electronics MG811 CO_2_ Sensor Features. https://sandboxelectronics.com/files/SEN-000007/MG811.pdf.

[25] Sensor Solutions (2017). MS5637-02BA03 Low Voltage Barometric Pressure Sensor. https://www.te.com/commerce/DocumentDelivery/DDEController?Action=showdoc&DocId=Data+Sheet%7FMS5637-02BA03%7FB1%7Fpdf%7FEnglish%7FENG_DS_MS5637-02BA03_B1.pdf%7FCAT-BLPS0037.

[26] Sparkfun (2023). Soil Moisture Sensor Hookup Guide. https://learn.sparkfun.com/tutorials/soil-moisture-sensor-hookup-guide/all.

[27] Arduino.cc (2023). Arduino Leonardo. https://docs.arduino.cc/hardware/leonardo.

[28] Winer K. Project Details for Hackable CMWX1ZZABZ (LoRa) Devices. 19 January 2018. https://hackaday.io/project/35169-hackable-cmwx1zzabz-lora-devices/details.

